# Health practitioners’ emotional reactions to caring for hospitalized children in Lomé, Togo: a qualitative study

**DOI:** 10.1186/s12913-017-2646-9

**Published:** 2017-12-04

**Authors:** Bassantéa Lodegaèna Kpassagou, Kokou Messanh Agbémélé Soedje

**Affiliations:** 1 University Hospital Campus of Lomé, Substitute teacher Faculty of Psychology, University of Lomé, Lomé, Togo; 2 University Hospital Campus of Lomé, Faculty of Health Sciences, University of Lomé, Lomé, Togo

**Keywords:** Health practitioners, Child patients, emotional stress, Psychological coping styles, Togo

## Abstract

**Background:**

Health practitioners frequently encounter dying, death and suffering. While providing health care can be stressful, the literature on how different health practitioners cope with the emotional challenges associated with their work is sparse. Further, much of this literature is based on studies conducted in high-income countries. In this study, we explored emotional distress and associated coping strategies among health practitioners working in a pediatric oncology department in a large teaching hospital in Lomé, Togo.

**Methods:**

We undertook a cross-sectional qualitative research involving in-depth interviews with 21 health practitioners (doctors, nurses, and nursing assistants) as well as facility-based observations Interview data were collected using a semi-structured discussion guide. All interviews were audio-recorded while observational notes were hand-written and ultimately typed. All data were transcribed, coded and analyzed thematically.

**Results:**

We found that practitioners experienced significant emotional distress. Their emotional distress was compounded by the seriousness of the illnesses they treated, the lack of appropriate medical equipment, and treatment failures that caused their patients to suffer. The health practitioners’ narratives suggested that a key reason for their emotional distress was a mismatch between their professional training and the realities of providing care in a resource-constrained setting. They also reported not receiving any training on how to cope with the emotional stresses associated with care and preventable patient deaths.

**Conclusion:**

Caring for patients is a source of significant emotional distress. The emotional stress experienced by health practitioners is compounded in resource-limited settings where weak health systems undermine practitioners’ ability to provide quality care. Results underscore the need to train health professionals to positively cope with the emotional stresses associated with patient care and for health systems improvements to ensure quality care.

## Background

Health practitioners frequently encounter dying, death and suffering. The emotional stress associated with these experiences can have negative effects on care providers. Jaffré and Olivier de Sardan’s [1] study of the relationship between health practitioners and patients in health facilities in five West African cities found a strong social distance between health practitioners and their patients, and widespread reports of poor quality care. Far from disparaging health practitioners, Jaffré and Olivier de Sardan argued that it was important to consider the psychological challenges that health practitioners face in the course of their work. Other studies similarly suggest that work stress, if prolonged and intensified, can result in chronic stress and affect the quality of care [2–4].

Caring for patients, particularly those with severe or terminal illness, can be emotionally taxing for health practitioners, resulting in feelings of powerlessness [5–7], and negatively affecting their wellbeing. Previous studies, for example, have reported posttraumatic stress [8, 9] and exhaustion [5] among health practitioners. Studies in France have also shown that psychiatric disorders are the leading cause of disability among practicing physicians [10] and that general practitioners have a suicide rate that is 2.5 times higher than the general population [11].

While the emotional challenges faced by health practitioners as a result of their work has received increasing attention in high-income countries [12], little is known about these issues in low and middle income countries. In Togo, where the current study was undertaken, the few studies on health practitioners focus on their experiences as medical professionals and/or on their job satisfaction [13, 14]. In this study, we explored the emotional strains faced by health practitioners working in a pediatric oncology department. We also assessed their strategies for coping with these work-related stressors.

## Methods

### Study setting

The study was conducted in the pediatric oncology department of a large teaching hospital in Lomé, Togo. The focus on health practitioners in the pediatric oncology department was informed by previous studies documenting the emotional challenges faced by facility-based oncology care providers [15, 16].

### Design and sampling

We conducted a cross-sectional qualitative study involving participant and facility observations of practitioner-patient interactions as well as individual interviews with health practitioners. All cadres of health practitioners practicing in the department were targeted. Health practitioners were eligible to participate if they were contractual employees of the study hospital. Interns, trainee nurses, and other temporary staff were excluded.

Twenty-one health practitioners (five doctors, nine nurses, and seven nursing assistants) consented to be interviewed. Their ages ranged from 24 years to 58 years, and averaged 38 years. Majority (62%, *n* = 13) were male. Eight participants had more than 5 years of professional employment and thirteen less than 5 years. Participants’ background characteristics are summarized in Table 1.

**Table 1:** Health practitioners’ characteristics (*N* = 21)

Variable		n
Sex	Male	13
	Female	8
Age (years)	23–34	6
	35–49	11
	50–59	4
Marital status	Single	4
	Married	14
	Divorced or separated	2
	Widower	1
Length of employment	Less than 5 years	13
	5–10 years	5
	More than 10 years	3

### Procedures

Data were collected between December 2015 and March 2016. We recruited participants by through a letter sent to all health practitioners in the child oncology care department. The letter clearly described the study and invited the providers to participate. Those who agreed to participate provided written consent. They were also assured that the researchers were not assessing the quality of clinical practice.

The participant observations were conducted by two researchers who were clinical psychologists and provided psychological support to oncology unit patients and their families. They interacted with ward staff and patients and took detailed notes of different patient-provider interactions. The research team observed 43 physicals examination, 26 resuscitation sessions, and 76 health assessment interviews. Details of the participant observations are reported elsewhere [17].

Following these observations, we conducted semi-structured individual interviews with all the consenting practitioners. The individual interviews focused on practitioners’ experiences in relation to the organization of work (working time, available equipment, relationship with the team and the hierarchy, and the organization of the staff); experiences relating to distressing emotional situations, such as patient deaths, and coping styles; and their suggested improvements. All interviews were conducted in French and audio-recorded.

### Analysis

Audio files and observational notes were transcribed. Two researchers independently read and re-read all the transcripts and created exploratory codes. Afterwards, the research team jointly developed a coding structure to manage transcribed data. [18–20]. We adopted a thematic analysis to explore key issues emerging from the data.

## Results

### Emotional difficulties of health practitioners

Participating practitioners’ narratives showed that the emotional challenges they face included: anger and frustration due to the lack of appropriate medical equipment and supplies to effectively manage patients, emotional stress of failing to successfully treat patients and of delivering “bad news” to the family of patients, and the lack of training on positive coping.

#### Anger and frustration due to the lack of medical equipment

Several practitioners reported that it was emotionally challenging to treat children with serious and chronic diseases because the hospital lacked appropriate medical equipment and supplies that would enable them provide quality care. Not being able to provide quality care to patients because of poor infrastructure reportedly resulted in anger, particularly among doctors. In what exemplifies the frustration and anger of providers as they worked without the right equipment and tools a pediatrician trying to resuscitate a six-year-old girl was observed saying:

*“There is really a problem of resources, but what can we do? The child is critically ill… we cannot even do a proper resuscitation! Is this a resuscitation session…? Is this resuscitation? Just see how it is bloating the child. The flask is filthy and in addition we push and release, we push and release… There is not even a respirator… this is not a resuscitation… they just have to leave the child to die peacefully… this is even disturbing her for nothing*”*.*

Practitioners regularly noted that the lack of medical equipment made them feel helpless and angry. In the words of one doctor, *“It’s hard to see the children suffer and die for by lack of resources because we are powerless against a solvable problem.”* Practitioners’ feeling of helplessness was compounded by the lack of equipment, delayed treatment, and prolonged patients’ suffering, which reportedly often made family members of patients feel that there was nothing the practitioners could do for their children. In the apt words of one doctor:

*“There are examinations that are not possible here…You wait, the results are not coming, you are forced to move, and during this time the child is suffering and you have no treatment, you can only say to them ‘we are expecting the results’…The disease advances and they have the impression that we cannot do anything for them. The parents get discouraged and want to go home but you cannot let them go, it's frustrating. Finally, you will learn that you are powerless in most cases.”*

The lack of quality medical equipment caused providers much emotional stress. Being unable to save lives, alleviate suffering, and restore good health among their patients made providers feel helpless and incompetent. Many of them reported being deeply pained, discouraged, and traumatized by seeing child who would, otherwise, have been successfully treated, suffer for long or die in their care. As one doctor put it: “*We are anyway powerless, so they die here and it is very rare for a family to thank us because most of them lose their children after spending a lot of time and money in the hospital.*” Emphasizing the feeling of discouragement and professional failure that follows poor treatment outcomes, one nurse noted that (health practitioners often feel that they were *“working for nothing.”*

#### Delivering “bad news”

Interview data also suggested that providers were often stressed by the experience of having to explain to parents about their child’s health, particularly in cases where the child had a terminal illness. While recounting a case involving a child whose cancer had metastasized, one doctor noted, “*The results we received showed that the cancer had already metastasized and there are no protocols here.... We cannot do anything. Then how do you tell this to a mother who only has one son... Really, this is not easy. These are the cases that I hate to do. It was the psychologist who handled this difficult situation.*” Emerging data showed that clinical psychologists were regularly asked to deliver news of severe or terminal illness to family members. They were also regularly asked to provide psychological support to patients and parents who were concerned about the quality of care.

#### Lack of training on positive coping

According to the practitioners we studied, their training did not adequately prepare them for the emotional distress associated with loss of life and prolonged patient suffering. The feeling of being ill-prepared to deal with the vagaries of providing care in a resource-poor context was described by a nurse: *“I wonder if my training allows me to have a distanced view on situations entrusted to me .... Did this training enable me to go into this domain? (...). Do I have the opportunity to accompany a person to the end of his or her life? I try to do my best to relieve the patients but do I really alleviate them?*”

### Coping with emotional distress

Judging by the data, practitioners coped with work-related emotional stress through avoidance and normalization of death.

#### Avoidance

Avoidance—or maintaining emotional distance from patients—was a strategy that practitioners commonly used to limit emotional distress. Practitioners noted that they often avoided emotional involvement with patients with advanced illness. This was particularly the case when practitioners felt that there was nothing they could do to help the patient given the lack of medical supplies and equipment to provide quality care. In the words of one nurse: “*When I see that a child has cancer in an advanced stage… when I expect that the child will die, my relationship with him or her is less close.”*

In some cases, practitioners’ inability to alleviate suffering led them to hope for the early death of the child. One provider’s experience of managing a terminally ill child was: *“... Afterwards, the first thing I did when I arrived at the hospital was to go find the dossier to see if the child didn’t pass away. It was a relief for the entire team when we knew the child was dead.*” Often, the death of suffering patients due to limitations of resources and equipment brought relief to providers.

#### Normalization and acceptance of death

Practitioners’ narratives also suggested that they coped with emotional distress by normalizing and accepting death. Illustrating the above point, providers noted *“Seeing somebody passing away doesn’t mean anything to me”, “I am armored, what is the death?”, “It is a daily thing and I don’t feel anything when I see someone die, the death, what is it?”,* and *“I feel nothing at all and I wait until it’s my turn.”*

Normalizing and accepting death as part of life was a coping strategy that developed over time as illustrated in expressions such as *“The most cancer deaths pass through here and we deal with it ... at the beginning it was difficult but now I'm armored”* and *“it is not affecting all nurses here, and some of them came and left after three weeks because they couldn’t stand to see people dying every day, but I'm here, it's been eight years since I [started] working here.”* Further, normalizing and accepting death was often reinforced by reflections about one’s own life as demonstrated below by a nursing assistant:

*There was a girl of ten years with a digestive tube cancer. She was in the CM1 class. To see that girl start crying and her parents in tears was not easy. He didn’t want to see this, she lamented, “I’m dying, I'm dying, I am in pain I am in pain ... I asked myself a lot of questions, I said to myself this could be my daughter. I have a daughter [who is] 10 years old and when I see cases like that I think of my daughter and I am afraid ... in the beginning I hid myself in the toilet and cried but then I said to myself, nurses do not cry”*

## Discussion

In this study, we explored care-related emotional strains faced by pediatric oncology care providers in Lomé, Togo and assessed how they coped with these strains. We found that providing care in this setting was emotionally challenging and that the emotional strains associated with caregiving were often compounded by the lack of effective medical treatment and equipment that made it difficult for practitioners to offer quality care.

Physicians, in particular, appeared to be vulnerable to feelings of anger and frustration due to the lack of medical supplies and equipment. Many of the participants emphasized that they felt helpless, discouraged, and anxious about being unable to provide optimal care for some patients. They also demonstrated low feelings of personal accomplishment and low self-efficacy. Similar findings have been reported in Tanzania, where health workers in primary health care facilities reported that the lack of laboratory equipment made them feel that they were “gambling” with patients’ lives [21]. Similarly, a study in Greece [22] showed that the reduction in public hospital budgets during the economic crisis resulted in medical supply shortages and was significantly associated with emotional exhaustion and low personal accomplishment among health practitioners. Working in resource-limited health settings may therefore compound the risk of emotional exhaustion and burnout for health workers.

Communicating bad news was the most difficult emotional task for many health practitioners. Barclay et al. [23] note that communicating bad news is stressful for health practitioners and that they experience strong emotions, such as anxiety, when they have to communicate such news. Unlike a previous study by Mansson and Kebede [24] who reported that doctors in Uganda and Ethiopia managed patients’ reactions by counseling them and giving them time to process the news, we found that clinical psychologists were regularly asked by physicians to communicate bad news. Arbabi et al. [25] found that physicians use different approaches to deliver bad news with some only involving family caregivers while others find it more appropriate to deliver the bad news directly to the patient. Health practitioners must be aware of patients’ emotional vulnerability and adopt empathetic ways to deliver bad news. In Togo, health practitioners receive only 12 h of training relationships between health practitioners and patients. Their limited training may lower their confidence in delivering bad news and result in their reliance on clinical psychologists. This culture of pluridisciplinarity in health care settings may be a useful way to use provide empathetic care to patients, particularly in cases of severe illness or death.

We found that avoidance as well as the normalization and acceptance of death were the most commonly reported strategies adopted to cope with the emotional stress associated with caregiving. In their study of coping strategies used by nurses in dealing with patient death and dying, Akuroma et al. [26] described normalization and acceptance of death as a psychological defense. Also described as the *“John Wayne syndrome”* (solitary cowboy and invulnerable to all emotion) [11], normalization and acceptance of death and dying is hinged on the faulty belief that good health practitioners are not ruffled by the suffering and death of patients [27]. However, experiencing death may make health practitioners mindful of their limitations and can be extremely painful and difficult [28]. These findings imply the need for better mental health support for healthcare professionals to manage the emotional stresses associated with caregiving. The findings also underscore the need for palliative care policies.

Study findings should, however, be interpreted with caution since we did not assess factors associated with different emotional reactions to caring. Further research to understand these factors is warranted. Understanding these factors can help identify strategies for alleviating emotional strains and ultimately improving health practitioners’ quality of life as well as the quality of services they provide.

## Conclusion

This qualitative study highlights the emotional strains faced by health practitioners in the course of providing care. Our findings show that caregiving can elicit strong feelings of anger and helplessness particularly in settings characterized by a lack of effective treatments and appropriate medical equipment. These results underscore the importance ensuring that health facilities are sufficiently resourced to ensure that health practitioners feel capable of providing the best quality care. In addition, results highlight the need to enhance health practitioners’ emotional coping skills during their professional training.
